# Drug Dependence Treatment Awareness among Japanese Female Stimulant Drug Offenders

**DOI:** 10.3390/ijerph13111127

**Published:** 2016-11-11

**Authors:** Shinzo Yatsugi, Koji Fujita, Saori Kashima, Akira Eboshida

**Affiliations:** 1 Department of Public Health and Health Policy, Institute of Biomedical and Health Sciences, Hiroshima University, 1-2-3 Kasumi, Minami-ku, Hiroshima 734-0037, Japan; white-eye@hiroshima-u.ac.jp (S.K.); karasu-karasu@hiroshima-u.ac.jp (A.E.); 2 Department of Medicine, Iwakuni Prison, 6-11-29, Nishimi, Iwakuni-city, Yamaguchi 741-0061, Japan

**Keywords:** stimulant drug, dependence, prevention, treatment, prisoners

## Abstract

Few stimulant drug users receive adequate treatment. This cross-sectional study describes the characteristics of female drug offenders that use stimulants and clarifies the factors related to the awareness of treatment for drug dependencies. We included 80 females imprisoned due to stimulant control law violations from 2012 to 2015. The characteristics of the female prisoners were stratified according to various treatment awareness levels, and associations between each characteristic and treatment awareness were evaluated using logistic regression models. The average period of stimulant drug use was 17.7 years. Participants imprisoned for the second time were significantly more likely to consider treatment compared to those imprisoned only once: odds ratio (OR) = 3.2 (95% confidence interval (CI): 1.0–10.7). This elevated OR was diluted in repeat offenders. Participants who had experienced multiple aftereffects (≥7) or serious depressive symptoms were also more likely to consider treatment: OR = 6.1 (95% CI: 1.8–20.8) and OR = 2.5 (95% CI: 1.0–6.2), respectively. Second-time stimulant offenders or offenders who had experienced health problems were more likely to consider it important to receive drug dependence treatment. To overcome relapses of stimulant use, it is recommended that stimulant use offenders are encouraged to accept adequate treatment.

## 1. Introduction

Illicit drug use is increasing, with an estimated 246 million global users in 2013 [1]. More than 1 in 10 drug users are found to suffer from drug-use disorders or drug dependence throughout the world [1], which can be a serious threat to public health systems and public health in general. The World Health Organization has labeled drug dependence as a sickness, and has highlighted the need to further strengthen public health systems to include treatments for drug-use dependence and associated disorders [2]. Unfortunately, it has been found that few drug users suffering from drug-use disorders or dependence receive adequate treatment [3], with only one in six receiving treatment in the world [1].

In East and Southeast Asia, stimulant drugs (e.g., amphetamines and methamphetamine) are widely used, of which crystal methamphetamine is the most popular drug among Japanese drug users [4,5]. Moreover, the primary method of drug use in Japan is via intravenous injection. After the promulgation of the Stimulant Control Law in 1951, stimulants (which include phenylaminopropanes, phenylmethylaminopropanes, as well as their salts and products containing them) were classified as illicit substances in Japan. Only designated medical care providers can administer stimulants to patients at a healthcare facility, but they cannot be prescribed for outside usage. As a result, stimulant users in Japan dramatically reduced after a peak of 55,664 arrests in 1954, but began to increase again in the 1970s [6]. Since 1998, the Japanese government has conducted five-year drug abuse prevention strategies in conjunction with updating their coordinated methods of eradicating substance abuse during each period (the fourth update occurred in August 2013) [7]. However, more than 10,000 people continue to be arrested for drug offenses each year [4]. Overall, while arrests for violating the Stimulant Drugs Control Act have decreased, repeat offenses have increased.

To prevent stimulant dependence and to allow users to successfully withdraw from the effects, it is important that the addict receives appropriate drug dependence treatment such as medication, a therapeutic community treatment, or cognitive drug dependence behavioral therapy [2,8,9]. However, many stimulant addicts have not obtained adequate treatment, despite having experienced methamphetamine psychosis symptoms or stimulant drug aftereffects. Those symptoms include consciousness disorders, hallucinations, delusions, or memory decline due to the strong pharmacological effect of stimulant drugs on the central nervous system (i.e., dopamine receptors) [6,10,11]. Although it has been recognized that treatment motivation is an important factor for drug abusers [8,12], there are limited studies regarding stimulant users due to the difficulties associated with collecting adequate samples.

In this paper, we examined the drug dependence treatment awareness of imprisoned female stimulant offenders and assessed the particular characteristics that may lead to the acceptance of drug dependence treatment.

## 2. Materials and Methods

### 2.1. Study Design and Participant Recruitment

A cross-sectional study design was used to collect samples from inmates of a female-only prison in Prefecture A from March 2012 to December 2015. The selected female prison had approximately 400 inmates in 2015, with approximately 60% having been imprisoned for the use of illegal drugs. Since 2006 all stimulant drug offenders in prison are obligated to participate in a guidance for overcoming drug dependence during their sentence, in accordance with the Act on Penal Detention Facilities and Treatment of Inmates and Detainees [13]. The program is a one-year course, and it commences within the first year of incarceration [14].

We targeted only new female prisoners that were incarcerated as a result of stimulant control law violations between March 2012 and December 2015 (*n* = 154), and selected the study sample based on the following inclusion criteria for inmates: (1) being above 20 years of age, because stimulant drug offenders who are younger than 20 years are usually referred to a Juvenile Training School; (2) having started participating in the guidance for overcoming drug dependence; and (3) having served less than two-thirds of their sentence (the remaining sentence was required to be longer than a year). We next selected 128 inmates who met the criteria and conducted a questionnaire survey. Among these samples, people who failed to understand the questions due to Intelligence Quotient (IQ) equivalent values of less than 69, and who were not in a condition to answer because of physical and mental conditions were excluded from the analysis. The purpose of the study was explained to those who agreed to participate, and informed consent was obtained. All information was corrected by a trained prison guard who is a law instructor for the Ministry of Justice. Law instructors have a background in education, social science, or psychology, and they are in charge of with correctional education by administrating Ministry training programs at various correctional institutions.

### 2.2. Measurements

#### 2.2.1. Individual Demographic Characteristics and the History of Substance Use

The predictive variables for each subcategory of behavioral and environmental factors (which are based on previous theories of health promotion [15]) were collected from the administrative dataset: (1) demographics and family characteristics (age, educational background, the presence or absence of employment prior to arrest, welfare beneficiary, IQ equivalence, and admission number); and (2) non-prescription/illicit substance use history (age at the first use of stimulant drugs, frequency of use during the 30 days prior to the arrest, period of stimulant use, major type of substances, and substance use of cohabitants). The IQ equivalence value was checked with the Correctional Association Psychological Assessment Series (CAPAS), which was developed for Japanese inmates [16]. A repeat offender in Japan was defined as an individual who had been admitted to prison three or more times, because some first offenders are granted suspended sentences and do not receive any correctional intervention. History of substance use was accessed at the time of inmate admission.

#### 2.2.2. Conditions of Withdrawal Symptoms and Self-Perceived Mental Health

Typical stimulant aftereffects were recorded when the participants received their first health examination in prison. In line with a previous study, nine withdrawal symptoms of stimulant aftereffects were identified in this study, including irritability, sensitivity to sound, tinnitus, insomnia, phlegmatic temperament, anxiety, psychomotor excitement, auditory hallucinations, and delusions [17]. All the symptoms were collected from a transmittal sheet of medical conditions, and the symptoms were diagnosed by a designated psychiatrist following an interview while in police custody or a detention house before imprisonment.

The information for the self-perceived mental health status was gathered before stating the guidance for overcoming drug dependence using a questionnaire based on the Addiction Severity Index—5th Edition [18,19]. The interviewer asked the participants yes/no questions, including “Have you experienced any of the following emotions within past 30 days before the arrest?” The items covered included: the experience of serious depression (associated with difficulties in daily functioning, sadness, hopelessness, or loss of interest); serious anxiety or tension (unreasonably worried or the inability to feel relaxed); hallucinations; trouble understanding, concentrating, or remembering; trouble controlling violent behavior, including episodes of rage or violence; serious thoughts of suicide (seriously considering a plan for taking their own life); and attempted suicide.

#### 2.2.3. Drug Dependence Treatment Awareness

In the guidance for overcoming drug dependence, drug dependence treatment awareness was examined from the question, “How important to you now is treatment for drug dependence?” Answers were given on a five-point Likert scale and classified into two categories: low (“not at all”, “slightly”, and “moderately”) and high (“considerably” and “extremely”).

### 2.3. Statistical Analysis

First, each characteristic for all of the study participants was described, and the participants’ characteristics were described and stratified according to the level of drug dependence treatment awareness. Differences between each characteristic for each awareness level were tested using the Kruskal–Wallis One-Way Analysis of Variance for continuous variables (quantitative variables) and a Pearson’s chi-square test for qualitative variables (category). Finally, the association between each categorical characteristic and each participant who felt that treatment was highly important was evaluated. Odds ratios (ORs) and the 95% confidence intervals (CIs) were calculated using logistic regression models. In this analysis, the following continuous variables were treated as categorical variables: age (10-year intervals), length of education (in years; <12 years indicated that they did not graduate from high school, or ≥12 years), IQ (tertile: ≤81, 82–89, and ≥90), number of times imprisoned (first time, second time, and repeat offenders), and the age at which they first used stimulant drugs (before graduating high school (≤18 years), and ≥19). In the evaluation of withdrawal symptoms, we also counted the number of symptoms. The number of withdrawal symptoms were treated as both continuous (one increment) and categorical variables (tertile: ≤4, 5–6, and ≥7). Since the participants primarily consisted of everyday users (88.8%) during the 30 days prior to the arrest, we excluded the variables of monthly stimulant-intake days in this logistic analysis.

Statistical analyses were performed using SPSS Windows 22.0 (SPSS Japan Inc., Tokyo, Japan). Probability (*p*) values less than 0.05 (two-sided) were considered to be statistically significant.

### 2.4. Ethics Approval

In accordance with the Private Information Protection Law, information that might identify the participants was safeguarded and deleted by the prison staff. This study was approved by the Ethics Committee of Epidemiological Research, Hiroshima University (#Epidemiology-1062), and written individual informed consent for anonymous participation in epidemiological research was obtained for each evaluation.

## 3. Results

Of the 128 study participants, 90 responded to the survey, indicating a 70.3% response rate. Of these, nine were excluded, as the CAPAS ability test revealed that their IQ equivalent values were less than 69, and one was excluded because the CAPAS was not conducted, leaving 80 participants for the final statistical analyses. The median period of detention on the interview day was three months (25th percentile: 2 and 75th percentile: 4), and the range was between 1 and 10 months.

Table 1 shows the characteristics of all the study participants, and those stratified by treatment awareness levels. Half of the participants considered drug dependence treatment to be considerably or extremely important.

### 3.1. Participant Characteristics

The mean age was 39.9 (standard deviation (SD): 8.8) years, with a range of 24–63 years. Three-quarters of the participants had less than 12 years of education, indicating that they did not graduate from high school. A quarter of the participants had been unemployed prior to their arrest, and half had been social welfare beneficiaries. The mean prison admission frequency was 2.4 times (SD: 1.7), and the highest was eight times. The proportion of repeated admissions was 36.3%, with more than 60% of the participants having been arrested more than twice.

In the subcategory of the history of non-prescription/illicit substances use, the average time of stimulant use was 17.7 (SD: 9.1) years. Of the participants, 88.8% had used a stimulant drug every day, and the mean days of stimulant intake in the 30 days prior to the arrest was 27.4 (SD: 7.6) days. More than 80% had used stimulant drugs with alcohol (63.8%) or other drugs (18.8%), and 58.8% had had housemates who were also substance users. All participants administered the stimulants via intravenous injection.

Approximately 86% of the participants had at least one stimulant drug aftereffect, and over 65% of the participants had withdrawal symptoms of irritability, sensitivity to sound, tinnitus, insomnia, phlegmatic temperament, and anxiety. A quarter of the participants had withdrawal symptoms consisting of auditory hallucinations or delusions. The average number of symptoms was 4.9 (SD: 2.8).

### 3.2. Participant Characteristics According to Treatment Awareness Levels

Differences in the participant’s characteristics between the lower and higher awareness levels were observed for the conditions of withdrawal symptoms and mental health status. The mean number of aftereffects were significantly higher for participants who considered treatment to be important than for those who felt that treatment was less important (*p* < 0.05). Participants who had the withdrawal symptoms of sensitivity to sound, tinnitus, insomnia, phlegmatic temperament, and auditory hallucinations were more likely to have high awareness levels of dependence treatment (*p* < 0.05). Higher awareness levels were also observed among patients who were affected by mental health problems, such as serious depression, severe anxiety or stress, and auditory/visual hallucinations.

There were no significant differences found for any of the other characteristics, including the demographic characteristics and a history of substance use. Of these, some participants who had a higher awareness for drug treatment were observed. The mean IQ score was lower for participants with the highest awareness levels (IQ = 84.2; SD: 8.1) compared with the mean score of those who had the lowest awareness (IQ = 86.0; SD: 8.9). The mean number of times the participants had been incarcerated was higher among the participants in the lowest awareness subgroup (2.5 times; SD: 1.9) compared to those in the highest awareness subgroup (2.3; SD: 1.4). For the participants who had had a substance-using cohabitant, they considered drug dependence treatment to be important.

### 3.3. Relationship between Prisoner Characteristics and High Treatment Awareness

Table 2 presents the relationships between the individual characteristics and participants who felt that drug dependence treatment was highly important. The participants who had been imprisoned a second time had a higher awareness of the necessity for treatment than the participants who had been imprisoned for the first time: OR of 3.2 (95% CI: 1.0–10.7). However, this elevated OR was diluted for the participants who were repeat offenders: OR of 1.1 (95% CI: 0.4–3.1). Furthermore, the participants who suffered from multiple aftereffects were found to be more likely to consider treatment: OR of 1.3 (95% CI: 1.1–1.5), with a one unit increment for each existing symptom. The ORs were 6.1 (95% CI: 1.8–20.8) for participants who reported seven or more aftereffects in the categorical analysis. Those associations were particularly observed among persons who had symptoms of drug aftereffects that consisted of sensitivity to sound, tinnitus, insomnia, phlegmatic temperament, and auditory hallucinations. For the self-perceived mental health status, participants who had suffered from serious depressive or anxiety symptoms in the 30 days prior to their arrest were more likely to exhibit a high treatment awareness: OR of 2.5 (95% CI: 1.0–6.2). A higher awareness was also observed among participants with auditory/visual hallucinations: OR of 3.1 (95% CI: 1.1–8.8).

## 4. Discussion

In this study, the characteristics of prison-based stimulant offenders were examined, and the relationship between participant characteristics and treatment awareness were evaluated. The results indicate that those participants who had been imprisoned for the second time or who had experienced health problems were highly likely to consider drug dependence treatment; however, repeat offenders (more than twice) were less likely to consider drug dependence treatment to be important.

The strengths of this study include the large sample of female prisoners that was used, of whom over 60% were repeat offenders due to a violation of the Stimulant Drugs Control Act, which was similar to the overall Japanese levels of repeat offenders in 2013 (63.8%) [4]. With repeated illicit drug use, a strong dependence typically occurs. Drug dependence is classified as physical dependence and psychological dependence. Physical drug dependence symptoms are easy to identify, and include trembling hands, sweating, diarrhea, and nausea. However, psychological drug dependence results in a decline in mental activity, making a recovery from drug dependence more difficult. Stimulant dependence has decreased or limited physical dependence symptoms, and is primarily associated with psychological dependence. If stimulant users are unable to recognize their physical symptoms as being related to their drug use, they have no fear of repeatedly using the drug, resulting in a higher tolerance and increased drug dependence. In addition, among stimulant users, a reverse tolerance from drugs has also been observed [20,21], in those with repeated use, psychopathic symptoms (e.g., auditory hallucinations and delusions) can develop from lower doses. Repeated stimulant use has been found to lead to poor physical health-related quality of life [22] and severe mental disorders [6,23,24,25]. To prevent repeated stimulant use, the perception of drug offenders toward drug dependence and treatment should be assessed in all prisons to illuminate the awareness of drug users.

In this study, 70% of second-time offenders considered treatment to be important. These findings are consistent with those of a previous study performed in the United States which reported high motivation among inmates who had been incarcerated more than once [26]. However, for repeat offenders (≥three times), the proportion of people with high awareness levels was only 45% in this study. Interestingly, the awareness of drug dependence treatment was found to decrease after the second offense; the more times an offender had reoffended, the lower their level of awareness. Although the higher awareness levels among repeat offenders in our study may already be aware of the treatment due to the experience of previous of incarnations, the awareness level decreased with the increased number of imprisonments after their second incarceration. They are presumed to fail repeatedly due to continued stimulant use, and they regress back into the precontemplation stage of the transtheoretical model [27]. This indicates that repeat offenders are more likely to lose the opportunity to receive treatment, meaning that they easily become repeat users following their release from prison. The longer the history of stimulant use, the more difficult it is to overcome the drug addiction; thus, even if the prison sentence was longer, it is still difficult to completely withdraw from the drug. After being released from prison without sufficient treatment, it is more likely that such individuals would begin taking the drug again [24,28]. Therefore, the correctional and treatment programs in prison should administer treatment to addicted inmates in accordance with each individual’s level of awareness or motivation, in accordance with the health promotion theory [27,29].

Relationships were also found between chronic health conditions and drug dependence treatment awareness levels. In this study, 86.2% of participants had at least one drug aftereffect symptom, which was similar to a previous study in Japan which reported drug aftereffect symptoms in 80%–90% of stimulant users [30]. Those who recognized the aftereffects exhibited high drug dependence treatment awareness levels, and participants with self-perceived mental health problems were also found to have high treatment awareness. If participants had experienced worsening withdraw symptoms or mental health conditions, they were more likely to feel strongly that their drug dependence should be treated. Therefore, therapy which combines drug dependence treatment with psychiatric treatment (e.g., medication and counseling) may be effective.

Although no significant differences between the prisoners’ IQ and treatment awareness were observed, those with a higher IQ were less likely to feel that treatment was necessary. Although there have not been any previous studies that have specifically focused on this aspect, there have been several reports showing that people with higher IQs tend to have stronger self-denial, and that the greater the self-denial, the higher the denial of the aftereffects, even if they are being experienced. Therefore, treatment is not often viewed as important. In such circumstances, there is less fear, leading to a greater attachment to the drugs. For offenders with higher than average IQs, this aspect of self-denial would need to be considered when discussing treatment options.

The limitations of this study are: (1) the motivation for treatment in this study was only measured from one questionnaire, due to the limited information associated with the administrative dataset in prison. Although the present findings may suggest a tendency toward greater/lower awareness levels of repeat offenders, more robust motivation scores—such as Circumstances, Motivation, Readiness (CMR) scores [31,32] or multiple questions in Simpson and Joe [26,33]—should be applied; (2) although all of the prison guards who collected the information from inmates were trained by the Ministry of Justice, there is a possibility of information bias in the collocational process with question-based surveys, especially regarding a mental health status that is based on the questions (which is not validated for the purpose of this study) in the addictive syndrome index. In addition, the mental health status in this study was related to the self-cognitive awareness of experienced symptoms. Thus, we could not generalize the present results to other mental health conditions which are diagnosed by clinical psychiatrists based on the Quick Inventory of Depressive Symptomatology, Diagnostic and the Statistical Manual of Mental Disorders fourth edition (DSM-4), or other clinical definitions; (3) the repeat offenders who presumably had participated in the guidance program during previous incarnations were contained. Since this may change the awareness levels of these prisoners, the effects of participating in the guidance on the awareness levels of repeat offenders should be evaluated in further studies; (4) the subjects were obtained from a single female prison, which may not be adequately large or diverse to generalize any of the observed relationships. Therefore, the present findings should be carefully interpreted. Although some ORs were not significantly elevated, positive ORs were observed. Future studies should include a larger sample population, as well as male stimulant offenders; and (5) first-time offenders exempted from prosecution and under a suspended sentence were not included in this study. In Japan in 2013, approximately 8.2% of the people arrested under the Stimulants Control Law were exempted from prosecution, and 39% of those who were indicted were administered suspended sentences [4]. Although the present findings cannot represent all first-time stimulant offenders, they can offer a perspective regarding the characteristics of repeated stimulant use in female offenders. As few studies have examined the importance of treatment and treatment awareness, this study makes a valuable contribution to the field.

Stimulant drugs are highly addictive, yet the denial of drug dependence means that encouraging treatment is extremely difficult [34]. Several previous studies have reported that the concentrated program is often not effective for stimulant users, because they are still within the precontemplation stage (approximately 40% of substance abusers) [35]. It is important for stimulant users to recognize drug dependence as a chronic health condition that requires long-term, continued treatment and care [1]. However, drug dependence treatment both globally and in Japan are unregulated [3]. In addition, at present, correctional programs in prisons have primarily focused on education but not treatment, and there are very few programs that focus on treating drug dependence [36,37]. The therapeutic community form of treatment [8,24] in an alternative incarceration program has not been installed within the Japanese system to date. Under these circumstances, after several years in prison, the methamphetamine psychosis (positive symptoms residual type) of inmates who have undergone insufficient or incomplete treatment has been found to increase [10,25], and inmates often lose their motivation for treatment. Appropriate prison-based treatment for drug users that utilize both pharmacological and psychosocial treatment modalities are required for each stage of the transtheoretical model [27,29]. Moreover, the treatment should be connected to an aftercare program after the prisoners’ release to reduce the chance of a re-offense.

## 5. Conclusions

Second-time stimulant offenders or stimulant offenders with health problems feel that receiving drug dependence treatment is important. However, it appears that if treatment is not offered, the belief in the importance of the treatment program is reduced for these prisoners. Therefore, it is important that in prison, adequate treatment is offered to stimulant offenders as soon as possible, and the awareness of the need for treatment should be increased for all stimulant offenders.

## Figures and Tables

**Table 1:** Study participant characteristics stratified by level of drug dependence treatment awareness.

		Total *n* = 80 (100%)	Awareness Level of a Drug Dependence Treatment ^1^		
			Low *n* = 40 (50%)	High *n* = 40 (50%)	*p*-Value
**Demographic**	Age, years (SD) *	39.9 (8.8)	39.8 (9.5)	40.1 (8.1)	0.86
	Education year length, *n* (%)				0.81
	<12 years	65 (81.3)	35 (53.8)	30 (46.2)	
	≥12 years	15 (18.8)	5 (33.3)	10 (66.7)	
	Employed before arrest, *n* (%)				0.11
	Unemployed	20 (25.0)	7 (35.0)	13 (65.0)	
	Employed	60 (75.0)	33 (55.0)	27 (45.0)	
	Social welfare recipient, *n* (%)				0.50
	Non-recipient	39 (48.8)	21 (53.8)	18 (46.2)	
	Recipient	41 (51.3)	19 (46.3)	22 (53.7)	
	IQ measured by CAPAS, points (SD) *	85.1 (8.5)	86.0 (8.9)	84.2 (8.1)	0.35
	Mean number of times in prison, years (SD) *	2.4 (1.7)	2.5 (1.9)	2.3 (1.4)	0.50
	Number of times in prison, *n* (%)				0.12
	First time	31 (38.8)	18 (58.1)	13 (41.9)	
	Second times	20 (25.0)	6 (30.0)	14 (70.0)	
	Repeated offense	29 (36.3)	16 (55.2)	13 (44.8)	
**History of substance use**	Age at first use of a stimulant, years (SD) *	19.3 (5.0)	19.2 (6.0)	19.4 (3.8)	0.89
	Stimulant intake during 30 days prior to the arrest, days (SD) *	27.4 (7.6)	27.1 (7.9)	27.6 (7.3)	0.76
	Period of stimulant use, years (SD) *	17.7 (9.1)	17.7 (10.4)	17.8 (7.7)	0.93
	Major type of substances, *n* (%)				0.23
	Stimulant drug only	14 (17.5)	8 (57.1)	6 (42.9)	
	Stimulant drug and alcohol	51 (63.8)	22 (43.1)	29 (56.9)	
	More than one drug	15 (18.8)	10 (66.7)	5 (33.3)	
	Cohabitant drug use, *n* of yes (%)	47 (58.8)	20 (42.6)	27 (57.4)	0.11
**Condition of withdraw symptoms at admission**	Having drug aftereffects at least one, *n* (%)	69 (86.3)	33 (47.8)	36 (52.2)	0.33
	Number of drug aftereffects, *n* of symptoms (SD) *	4.9 (2.8)	4.0 (2.7)	5.8 (2.6)	0.00
	Symptom of drug aftereffects, *n* of yes (%)				
	Irritable	65 (81.3)	31 (47.7)	34 (52.3)	0.39
	Sensitivity to sound	52 (65.0)	19 (36.5)	33 (63.5)	0.00
	Tinnitus	52 (65.0)	20 (38.5)	32 (61.5)	0.00
	Insomnia	56 (70.0)	23 (41.1)	33 (58.9)	0.01
	Phlegmatic temperament	62 (77.5)	27 (43.5)	35 (56.5)	0.03
	Anxiety	63 (78.8)	30 (47.6)	33 (52.4)	0.41
	Psychomotor excitement	3 (3.8)	0 (0.0)	3 (100.0)	0.08
	Auditory hallucinations	20 (25.0)	5 (25.0)	15 (75.0)	0.01
	Delusion	19 (23.8)	6 (31.6)	13 (68.4)	0.07
**Mental health status**	Self-perceived status, *n* of yes (%)				
	Serious depression	43 (53.8)	17 (39.5)	26 (60.5)	0.04
	Severe anxiety or stress	43 (53.8)	17 (39.5)	26 (60.5)	0.04
	Auditory/visual hallucinations	23 (28.8)	7 (30.4)	16 (69.6)	0.03
	Memory deterioration	20 (25.0)	9 (45.0)	11 (55.0)	0.22
	Psychomotor excitement	20 (25.0)	9 (45.0)	11 (55.0)	0.61
	Suicidal ideation	26 (32.5)	11 (42.3)	15 (57.7)	0.34
	Attempted Suicide	18 (22.5)	9 (50.0)	9 (50.0)	1.00

Note: *p*-values were calculated using Pearson’s Chi-Square Test and Kruskal–Wallis One-Way Analysis of Variance (*); SD, standard deviation. ^1^ The awareness levels were categorized as low (“not at all”, “slightly”, and “moderately”) and high (“considerably” and “extremely”). CAPAS, Correctional Association Psychological Assessment Series; IQ, intelligence quotient.

**Table 2:** Relationship between each characteristic and a high drug dependence problem awareness (*n* = 80).

		Total *n* (%)	OR ^1^	(95% CI)
**Demographic**	Age			
	≤29 years	10 (13)	Ref.	
	30–39	31 (39)	1.4	(0.3–6.0)
	40–49	30 (38)	2.0	(0.5–8.4)
	≥50	9 (11)	1.2	(0.2–7.4)
	Education year length			
	<12 years	65 (81)	Ref.	
	≥12 years	15 (19)	2.3	(0.7–7.6)
	Employment status before arrest			
	Employed	60 (75)	Ref.	
	Unemployed	20 (25)	2.3	(0.8–6.5)
	Social welfare recipient			
	Non-receipt	39 (49)	Ref.	
	Recipient	41 (51)	1.4	(0.6–3.3)
	IQ measured by CAPAS (tertile)			
	≥90	24 (30)	Ref.	
	82–89	29 (36)	1.1	(0.4–3.2)
	≤81	27 (34)	0.9	(0.3–2.8)
	Number of times in prison			
	First time	31 (39)	Ref.	
	Second times	20 (25)	3.2	(1.0–10.7)
	Repeated offense	29 (36)	1.1	(0.4–3.1)
**History of substance use**	Age for first stimulant drug use			
	≤18 years	46 (58)	Ref.	
	≥19	34 (43)	1.5	(0.6–3.7)
	Period of stimulant use			
	≤10 years	21 (26)	Ref.	
	11–19	34 (43)	2.1	(0.7–6.3)
	≥20	25 (31)	1.8	(0.5–5.7)
	Major ingested substances			
	Stimulant drug only	14 (18)	Ref.	
	Stimulant drug and alcohol	51 (64)	1.8	(0.5–5.8)
	More than one drug	15 (19)	0.7	(0.1–3.0)
	Drug use of cohabitant			
	No	33 (41)	Ref.	
	Yes	47 (59)	2.1	(0.8–5.1)
**Condition of withdraw symptoms at admission**	Symptom of drug aftereffects ^2^			
	Irritable	65 (81)	1.6	(0.5–5.2)
	Sensitivity to sound	52 (65)	5.2	(1.9–14.5)
	Tinnitus	52 (65)	4.0	(1.5–10.8)
	Insomnia	56 (70)	3.5	(1.2–9.7)
	Phlegmatic temperament	62 (78)	3.4	(1.1–10.6)
	Anxiety	63 (79)	1.6	(0.5–4.7)
	Psychomotor excitement	3 (4)	NE	
	Auditory hallucinations	20 (25)	4.2	(1.4–13.1)
	Delusion	19 (24)	2.7	(0.9–8.1)
	Having drug aftereffects (at least one)			
	No	11 (14)	Ref.	
	Yes	69 (86)	2.1	(0.8–5.1)
	Number of drug aftereffects			
	Continuous (1 increment)		1.3	(1.1–1.5)
	Category (tertile)			
	≤4	31 (39)	Ref.	
	5–6	28 (35)	3.3	(1.1–9.6)
	≥7	21 (26)	6.1	(1.8–20.8)
**Mental health status**	Self-perceived status ^2^			
	Serious depression	43 (54)	2.5	(1.0–6.2)
	Severe anxiety or stress	43 (54)	2.5	(1.0–6.2)
	Auditory/visual hallucinations	26 (33)	3.1	(1.1–8.8)
	Memory deterioration	23 (29)	1.9	(0.7–5.0)
	Psychomotor excitement	20 (25)	1.3	(0.5–3.6)
	Suicidal ideation	26 (33)	1.6	(0.6–4.1)
	Attempted Suicide	18 (23)	1.0	(0.4–2.9)

OR, odds ratio; CI, confidence interval; NE, not estimable; Ref., reference; CAPAS, Correctional Association Psychological Assessment Series; IQ, intelligence quotient. ^1^ OR for people who felt the treatment program was extremely or considerably important for each characteristic; ^2^ People with no symptoms were referenced for each symptom and duration.
